# Spray in Ovariotomy

**Published:** 1885-03

**Authors:** John Homans

**Affiliations:** 161 Beacon Street, Boston


### SPRAY IN OVARIOTOMY.

*Editors of the Atlanta Medical and Surgical Journal:*

The following is an extract from a recent work by Dr. Emmet: (Emmet's Principles and Practice of Gynæcology, p. 715):

"In this country I do not know of any prominent operator who now employs the carbolic acid spray." This statement implies that the writer is not persuaded of the value of spray in ovariotomy. My own experience has led me to an opposite opinion. Indeed I should not like to do a laparotomy for any purpose without antiseptic spray. I have been led to this conclusion by the results of one hundred and eighty-three cases of removal of cystic ovaries, of which I have lost only twenty-one, but more especially by the result of the last one hundred of these cases, only ten of which were fatal, while thirty-eight were consecutively successful. I feel that to omit the antiseptic spray would be to deprive the patient of one of the ready and efficient elements of success. As I can hardly hope for much better results than those I have cited, and being quite content to let well alone, I shall hesitate before disturbing my present plan of operation by giving up a detail to which I attach much importance.

I am very respectfully your obedient servant,

JOHN HOMANS.

*161 Beacon street, Boston, Feb. 17, 1885.*

